# Efficient optimization of R50% when planning multiple cranial metastases simultaneously in single isocenter SRS/SRT

**DOI:** 10.1002/acm2.13254

**Published:** 2021-05-07

**Authors:** Dharmin D. Desai, Ivan L. Cordrey, E.L. Johnson

**Affiliations:** ^1^ Department of Radiation Oncology CHI Memorial Hospital Chattanooga TN USA; ^2^ Department of Radiation Medicine University of Kentucky Chandler Medical Center Lexington KY USA

**Keywords:** optimization constraints, R50%, single isocenter multiple metastases, SRS

## Abstract

Simultaneous optimization of multiple Planning Target Volumes (PTVs) of varying size and location in the cranium is a non‐trivial task. The rate of dose falloff around PTV structures is variable and depends on PTV characteristics such as the volume. The metric R50% is one parameter that can be used to quantify dose falloff achieved in a given treatment plan. An important treatment planning question is how to construct optimization conditions that result in the efficient production of acceptable plan outcomes considering metrics such as R50%. Guidance provided in literature suggests generating multiple shell control structures around each PTV. The constraints applied to these shells can vary significantly depending on PTV volume. Additionally, there is no clear guidance on how to prospectively determine objective constraints for the optimization shells to achieve a specified goal of R50%. Based on physical principles and empirical evidence, we provide clear quantitative guidance on how to translate the desired R50% outcome into appropriately sized optimization structures around PTVs via an equation that depends on a desired goal for R50% and the volume of PTV. Optimization schema are also provided that allow the goal R50% to be approached or achieved for all PTVs individually. We demonstrate the application of the methodology using commercially available treatment planning software and radiotherapy treatment equipment.

## 1 INTRODUCTION

Stereotactic Radiosurgery (SRS) and Stereotactic Radiotherapy (SRT) aim to deliver high doses of ionizing radiation to cranial targets with high precision. The application of linac‐based SRS/SRT in the treatment of brain metastases has become increasingly common in the Radiation Therapy clinic. Early approaches in the application of SRS/SRT for brain metastases were limited to single targets or to the treatment of multiple targets utilizing a multi‐isocenter technique. Multiple targets were treated sequentially requiring setup changes and verification for each isocenter. Recent advances in treatment delivery technology and planning have allowed the simultaneous targeting of multiple cranial targets using a single isocenter.^1^, ^2^ This approach results in efficient workflows and decreased treatment times. Volumetric Modulated Arc Therapy (VMAT) techniques such as RapidArc and its implementations within HyperArc^3^ and RapidPlan^4^ (Varian Medical Systems, Palo Alto, CA) have produced efficient delivery schemes, improved target coverage and/or reduced normal tissue doses. However, sparing normal tissues within the cranium is a necessary goal that often further complicates the treatment planning process. The single isocenter approach for targeting multiple sites simultaneously has increased the required treatment planning effort needed to produce acceptable dose distributions, especially when considering normal tissue objectives (NTO). Ballangrud et al. comment on the increased treatment planning effort required in the statement; “To further improve VMAT planning for multiple cranial metastases, better tools to shorten planning time[s] are needed.”^5^

The cranium contains many normal structures requiring consideration in planning optimization such as the brainstem, optic chiasm, and optic nerves. Minimizing normal brain tissue dose is also an important optimization objective as it is always directly adjacent to Planning Target Volume (PTV) surfaces and subject to the high doses being delivered to these PTVs. Indeed, radiation necrosis of normal brain tissue is one of the more relevant adverse effects after SRS/SRT.^6^ Various publications have evaluated the intermediate dose spill from PTV surfaces using metrics such as V12 Gy (the volume of brain receiving 12 Gy) and have identified potential complications associated with excessive volumes for these quantities.^7^, ^8^, ^9^ These studies stress the need to minimize dose spill metrics to reduce normal brain tissue doses and the incidence of associated complications. A widely accepted approach used to control intermediate dose spill uses multiple, contiguous shells surrounding the PTVs.^2^, ^10^, ^11^, ^12^ Typically, three shells are defined that step‐down dose in a controlled manner. The size and dose constraints on these shells can be dependent on the PTV size. More importantly, initial conditions applied to these shell structures do not necessarily translate into acceptable intermediate dose spill values. When targeting multiple PTVs using a single isocenter, the multiple fixed shell approach can be susceptible to unacceptable dose falloff requiring adjustment of shell parameters and repeat optimizations adding to the treatment planning effort. In addition, no clear guidance on shell parameter modification is available.

Various metrics have been devised to quantify and potentially control this intermediate dose spill. The metric R50% is the parameter we utilize in this work to develop schema to control intermediate dose spill from the PTV surface. R50% is defined as the 50% isodose cloud volume (V_IDC50%_) normalized by the PTV volume (V_PTV_).^13^ Thus:

(1) $$\text{R50}\%=\frac{V_{\text{IDC50}\%}}{V_{\text{PTV}}}$$

An alternative intermediate dose spill metric commonly used metric in SRS planning when evaluating competing plans is Gradient Index (GI). Paddick defines GI as the ratio of V_IDC50%_ to the 100% isodose cloud volume (V_IDC100%_).^14^ Clearly, if the plan is perfectly conformal in the high dose region, V_IDC100%_ is equivalent to V_PTV_, and GI is equivalent to R50%. However, if V_IDC100%_ is not perfectly conformal to the PTV, plan flaws can be masked. For example, a V_IDC100%_ larger than V_PTV_ is possible, and, in such a case, GI would not adequately account for the normal tissue that falls within V_IDC100%_ but outside the PTV surface. A plan with an acceptable GI could consequently be an inferior plan in terms of the normal tissue outside of the PTV being radiated to a high dose. In a study of LINAC based RapidArc SRS plans, such a phenomenon was identified where the RapdiArc plans appeared to have noticeably larger GI values compared to Gamma Knife plans.^15^ Liu et al. state, “The larger GI values for the RapidArc SRS plans are not because they have larger 50% prescription isodose volume but because they all have smaller 100% prescription isodose volume.” In other words, the larger GI results from being more conformal in the high dose region. This counterintuitive behavior of GI is avoided with R50% because it is defined directly in terms of V_PTV_. Using R50%, we have devised a more robust, efficient, and better‐defined method for controlling intermediate dose spill that does not typically require iterative optimization, thus reducing treatment planning time required for these single isocenter, multiple PTV techniques.

A good multiple target cranial SRS/SRT treatment must have a clinically efficient beam delivery geometry. Many authors have described how to achieve such delivery geometries with a single isocenter approach.^1^, ^2^, ^16^, ^17^ Furthermore, the treatment plan must have reasonable optimization goals against which to assess the outcome and determine when an acceptable plan has been achieved. Several authors have suggested such planning goals and even some strategies to achieve those goals.^2^, ^12^, ^18^ Given an optimal treatment geometry and a well‐specified optimal planning goal, what has been missing is a concise optimization approach for achieving that final goal. This work addresses that third issue – the concise optimization approach to achieve the final goal, at least as it applies to R50%. Note: A table of abbreviations is provided in Appendix A.

## 2 METHODS

### 2.1 “Ask For It” Approach for R50%

The “Ask For It” (AFI) inverse planning approach is a two‐step process. The first step is the construction of an optimization shell specifically dependent on the volume of the PTV and the R50% goal one wishes to achieve. The second step is the prospective determination of the optimizer volumetric constraint dependent on the R50% goal, the PTV volume, and the optimization shell volume. As such, one is able to explicitly ask the optimizer for the desired R50% goal final result – we ask for R50%_Goal_. Below is the summary of our empirically determined AFI approach. The detailed derivation and articulation of the approach are given in the Appendix B.

Given a R50% goal (R50%_Goal_), we construct a unique optimization shell and inverse planning optimization criterion customized for each PTV. The unique optimization shell scales to the characteristics of the individual PTV and the specified R50%_Goal_. The exact nature of the R50%_Goal_ is independent of the AFI approach, so as our understanding of the appropriate R50%_Goal_ changes and improves, the AFI approach does not change.

**Fig. 1:**
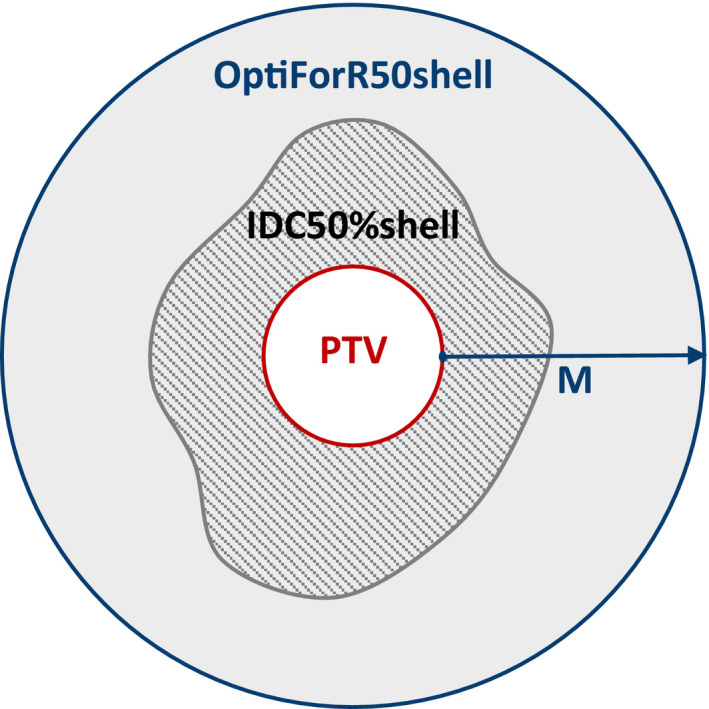
Simplified anatomy of the optimization structure surrounding the PTV. OptiForR50shell is generated by expanding the PTV by a value of M and includes everything within its boundary (gray shaded area) except for the PTV. IDC50%shell is the patterned area within the gray shaded area (IDC50%shell = IDC50% ‐ PTV).

As an idealized case, consider a spherical PTV with volume V_PTV_ surrounded by an isodose cloud of 50% of the prescription (Rx) dose (IDC50%) as illustrated in Fig. 1. Define an isodose shell (IDC50%shell) bounded externally by IDC50% and internally by the PTV surface. Construct an optimization structure encompassing the IDC50%shell called OptiForR50shell that is large enough that the final ICD50%shell based on the R50%_Goal_ occupies approximately 20% of the OptiForR50shell. The volumetric condition 20% is not a rigid requirement but was determined empirically to provide a stable solution for various PTV shapes and sizes. In practice, we have found that the value varies from 15% to 20%; this is explicitly calculated in the AFI approach. The expansion margin, M, to be applied to the PTV that provides the approximate 20% volumetric requirement for OptiForR50shell is given by:

(2) $$M={\left(\frac{3}{4\pi }V_{\text{PTV}}\right)}^{1/3}\times \left[{\left(5\times R50{\%}_{\text{Goal}}‐4\right)}^{1/3}‐1\right]$$

where V_PTV_ is in units of cm^3^. M is given in cm and depends on two important factors, V_PTV_ and R50%_Goal_. Expanding the PTV by the margin M creates the outer surface of a structure called OptiForR50. OptiForR50shell is the difference between OptiForR50 and the PTV and has a volume V_OptiForR50shell_. Next, we have to determine the fractional percentage of OptiForR50shell that should receive 50% of the prescription dose (%V_Opti_) to achieve the R50%_Goal_. This value is given by:

(3) $$\%V_{\text{Opti}}=100\times \frac{\left(R50{\%}_{\text{Goal}}‐1\right)\times V_{\text{PTV}}}{V_{\text{OptiForR}50\text{shell}}}$$

The above procedure is applied separately for each individual PTV. As such, the OptiForR50shell and the %V_Opti_ is unique to each PTV and is dependent on the R50%_Goal_.

Finally, a single global structure that is outside all the PTVs and OptiForR50shell structures is constructed, which we call iShell. This is an insurance shell with the only purpose to insure that the 50% isodose cloud remains within each individual OptiForR50shell. The structure Boolean algebra expression for iShell is:

(4) $$\text{iShell}=\text{Brain}\;‐\sum_{\text{i = 1}}^{n}\text{(PTV}+{\text{OptiForR50shell)}}_{i}$$

where the summation is over the n number of PTVs within the cranium.

It is important to note that the AFI approach described above would only need to be performed one time with this one set of optimization parameters; multiple iterations are typically not required.

### 2.2 R50%_Goal_ determination

The AFI approach summarized in section 2.A and derived in detail in Appendix B depends on having a goal for R50%, the R50%_Goal_. This R50%_Goal_ is currently not a settled question, but some guidance has been published.^2^, ^5^ In this work, we take our R50%_Goal_ from reprocessing information in the work of Ballangrud et al. where the authors provide a phenomenological fit for GI in terms of the V_PTV_ (GI = 4V_PTV_^‐0.2^) for plans that have been determined to be optimal. In addition, the mean Conformity Index (CI) is reported for the plans assessed (CI = 1.2 ± 0.1). Considering the product of GI and CI:^14^

(5) $$\text{GI}\times \text{CI}=\frac{V_{\text{IDC50}\%}}{V_{\text{IDC100}\%}}\times \frac{V_{\text{IDC100}\%}}{V_{\text{PTV}}}=\frac{V_{\text{IDC50}\%}}{V_{\text{PTV}}}=\text{R50}\%$$

Therefore, these data of Ballangrud et al. can be used to estimate a planning goal for R50%_Goal_.

(6) $$\text{R50}{\%}_{\text{Goal}}=\text{GI}\times \text{CI}=4\text{.8}{\left(V_{\text{PTV}}\right)}^{‐0\text{.2}}$$

The R50%_Goal_ values obtained from Eq. (6) are used in Eqs. (2) and (3) to determine the critical parameters for implementing the AFI approach.

### 2.3 Phantom studies

**Fig. 2:**
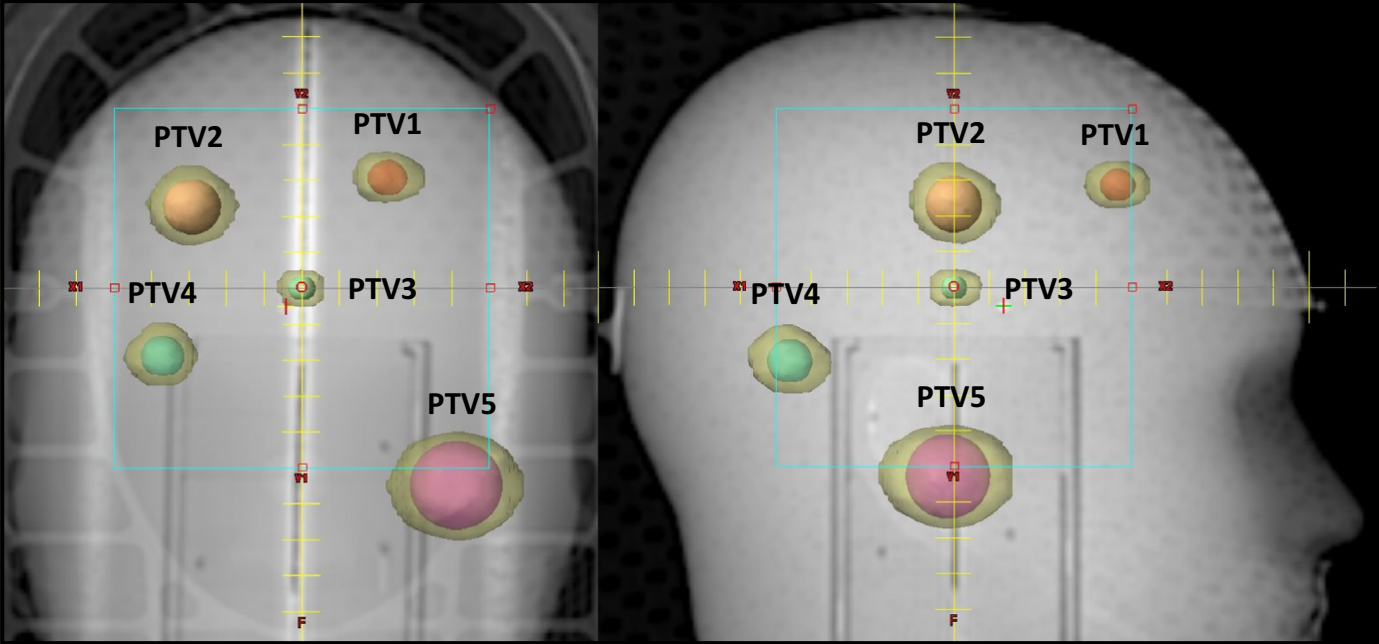
AP and LAT DRR views showing the locations of the five Spherical PTVs. The center locations of the irregular and jack shaped PTVs are similar to those shown for the spherical PTVs; only the size and shape change. The final IDC50% volumes are shown in transparent yellow around each spherical PTV.

The 3D anthropomorphic patient model used in the study was obtained from a treatment planning CT of the IROC Head Phantom® (IROC Houston QA Center, Houston, TX). We ignored the IROC PTV and created a unique set of 5 PTVs (PTV1 ‐ PTV5) distributed throughout the cranial cavity. Three planning scenarios were constructed using different PTV shapes, sizes, and locations. The first, Plan 1, utilized 5 spherical PTV volumes ranging from 0.19 cm^3^ to 8.0 cm^3^ as Illustrated in Fig. 2. Plan 2 used 5 irregularly shaped PTVs, while Plan 3 utilized 5 jack‐shaped PTVs. The unique center coordinates for the 5 PTVs were kept similar in all three plans, only the shape and size of the PTV changed. The term “jack” refers to a 3D solid composed of three orthogonal ellipsoids sharing a common center. This shape resembles the six‐pointed “Jacks” game piece. Thus, the three ellipsoids of the jack are oriented with their longest axes in different directions: superior‐inferior, anterior‐posterior, and right‐left. These jacks represent a combination of extremely concave and convex shapes (at the tips of the ellipsoids). Although the jack shape is not likely for a clinical PTV, it represents an extremely nonspherical case that tests the PTV shape limits of our AFI approach. The PTV characteristics are summarized in Table 1.

**Table 1:** PTV volumes used in the planning/optimization study.

Structure	Plan 1 Spherical Volume (cm^3^)	Plan 2 Irregular Volume (cm^3^)	Plan 3 Jack Volume (cm^3^)
PTV1	0.54	1.20	1.50
PTV2	1.96	0.98	1.44
PTV3	0.19	0.73	1.11
PTV4	0.97	1.01	1.83
PTV5	8.00	1.67	1.28

The Rx dose utilized for all optimizations and final plans in this study is 9 Gy x 3 fractions for a total dose of 27 Gy. Optimizations were performed on an Eclipse Treatment Planning System (TPS) (Varian Medical Systems, Palo Alto CA) v 15.6 using the optimization algorithm PO v 15.6. Optimization parameters used for a typical PTV are summarized in Table 2. There would be a similar set of unique parameters applied for each of the PTVs in the multiple target case. The iShell objectives would only be declared once since it is a single global structure. The automatic Normal Tissue Objective (NTO) option was also used as an optimization condition,^12^, ^19^ although this may not be necessary since, in any clinical case, one would also have dose limits for specific Organs At Risk (OAR), e.g., brainstem, optic chiasm, cochlea, etc., based on standard protocols.^20^ However, these are beyond the scope of this study and are not included in this optimization.

**Table 2:** The optimization parameters and relative penalties for implementing the AFI strategy for SRS/SRT multiple target plans.

Structure	Volume (%)	Min Dose (% of Rx)	Max Dose (% of Rx)	Penalty (relative number)
PTV	0	‐	140	200
	100	100	‐	200
OptiForR50shell	0	‐	100	200
	%V_Opti_	‐	45	200
iShell	0	‐	45	200

There will be a unique set of such shells and optimization parameters for each PTV in a multiple target case. OptiForR50shell is a shell expansion of the PTV with the expansion margin given by M in Eq. (2), and %V_Opti_ is given by Eq. (3), and both parameters are dependent on the R50%_Goal_ specific to that PTV. The iShell is a single global structure defined by Eq. (4), which is the brain minus all the PTVs and OptiForR50shell. The penalties listed are a defined part of the AFI approach but may need minor modifications for planning systems other than Eclipse.

Plans were generated for each of the three PTV sets described above. All plans utilized a single, centrally located isocenter with multiple RapidArc® VMAT ARCs and 6 MV photons. The collimator angles were chosen to minimize situations in which two targets fell in the same leaf track to discourage open leaves between targets and thus unnecessary dose spill. Treatments were designed for delivery on a Varian TrueBeam LINAC configured with a 120 leaf Millennium MLC (Varian Medical Systems, Palo Alto, CA). The beam geometry used for each case is summarized in Table 3. Final dose calculations were performed using the AAA v15.6 algorithm on a 1 mm calculation grid size. After final dose calculation, the PTV volumetric dose coverage was assessed for each PTV. The PTV least covered by the prescription dose was used to renormalize the entire plan and to ensure that every PTV has at least 95% of its volume covered by at least the full prescription dose (D95% Rx).

**Table 3:** Beam Delivery Geometry for treatment planning studies.

PTV geometry	No. of targets	No. of arcs	Collimator angles	Table angles	Arc lengths	MUs
Spherical	5	6	85, 15, 5,	0, 0, 0,	350, 350, 350,	6547
			85, 95, 30	90, 90, 90	170, 170, 170	
Irregular	5	6	85, 45, 85,	0, 0, 0,	350, 350, 350,	8143
			85, 95, 45	90, 90, 90	170, 170, 170	
Jack	5	6	85, 15, 85,	0, 0, 0,	350, 350, 350,	7684
			85, 95, 15	90, 90, 90	170, 170, 170	

### 2.4 Plan assessment using OptiForR50shell

Another challenge of a multiple target SRS/SRT case is how to efficiently evaluate the plan quality for each PTV independently to ensure that the dose coverage and drop‐off for each PTV is optimal. This can be achieved with the creative use of the same structures used in the optimization.

The CI requires the determination of the volume of the 100% of Rx dose (V_100%_) for each target independently. We designate an arbitrary individual PTV as PTV_n_ and let the subscript n propagate through all structures derived from PTV_n_. One cannot obtain the individual V_PTVn100%_ value or each PTV_n_ directly from the TPS since the Dose Volume Histogram (DVH) tools would return a cumulative value that includes every target. However, if one defines:

(7) $$\begin{array}{c} \%\text{V1}00{\%}_{\text{PTVn}}\\ \;=\%{\;\text{of PTV}}_{n}\text{that received at least the Rx dose for target n} \end{array}$$

and

(8) $$\begin{array}{c} \%\text{V1}00{\%}_{\text{OptiForR5}0\text{shelln}}\\ \;=\%\;\text{of OptiForR5}0{\text{shell}}_{n}\text{that received at least the Rx dose for target n} \end{array}$$

then

(9) $${\text{CI}}_{n}=\frac{\left[\left(\%V100{\%}_{{\text{PTV}}_{n}}\times V_{\text{PTV}}\right)+\left(\%V100{\%}_{\text{OptiForR}50{\text{shell}}_{n}}\times V_{\text{OptiForR}50{\text{shell}}_{n}}\right)\right]}{V_{{\text{PTV}}_{n}}}$$

The first term in the numerator defines the amount of volume of the 100% Rx dose cloud that falls inside the PTV; the second term defines the amount of volume of the 100% Rx dose cloud spills outside the PTV. All four quantities in Eq. (9) can be conveniently read from the DVH and the structure statistics provided by the TPS or extracted by a script.

Similarly, R50%_n_ can be acquired for each PTV individually. Define:

(10) $$\begin{array}{c} \%\text{V5}0{\%}_{\text{OptiForR5}0\text{shelln}}\\ \;=\%\;\text{of OptiForR5}0{\text{shell}}_{n}\text{that received at least 5}0\%\;\text{Rx dose for target n} \end{array}$$

Then:

(11) $$\text{R50}{\%}_{n}=\frac{\left[\left(\%\text{V50}{\%}_{{\text{OptiForR50shell}}_{n}}\times V_{{\text{OptiForR50shell}}_{n}}\right)+V_{{\text{PTV}}_{n}}\right]}{V_{{\text{PTV}}_{n}}}$$

The numerator is the total volume of the IDC50% cloud. This analysis was used to assess quality of the final optimized plans for each target individually.

### 2.5 Plan QA

It is important that the plans created with the AFI approach be clinically deliverable and meet reasonable quality assurance standards. Thus, each of the three plans was delivered and quality was assured using an ArcCHECK® (Sun Nuclear, Melbourne, FL), with all couch angles set to 0°. Evaluation criteria of 3%, 2 mm DTA, and a 10% dose threshold were utilized to determine passing rates.

## 3 RESULTS

**Fig. 3:**
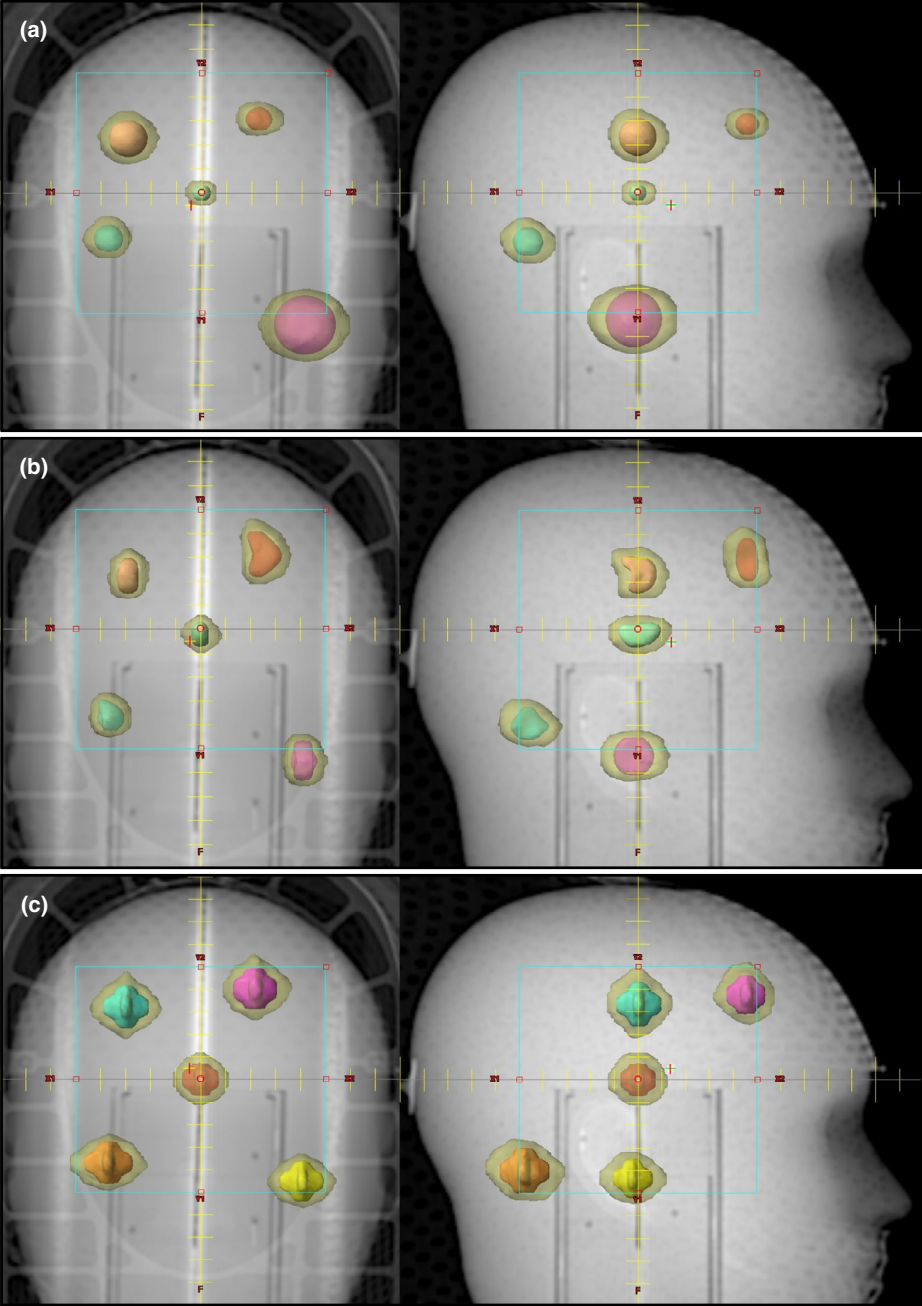
The final results of a single optimization run using the AFI approach. Shown are the five PTVs (solid color) of each multi‐target plan and the IDC50% (transparent yellow). All plans use a single isocenter, two couch angles, and six arc delivery to treat all PTVs simultaneously. The AP and right lateral setup field is shown for scale. (a) Five spherical shaped PTVs of widely varying volumes, 0.19 cm^3^ to 8 cm^3^. (b) Five irregularly shaped PTVs of volumes 0.73 cm^3^ to 1.67 cm^3^. (c) Five jack shaped PTVs of volumes 1.11–1.83 cm^3^. Notice there are no extraneous IDC50% volumes far from the PTVs, and any given individual IDC50% is highly conformal and of comparable shape to its associated PTV.

The final results for plans optimized by the AFI approach are shown visually in Fig. 3. This is the product of the first and only optimization run – it is not an iterative optimization. Here, one can see the geometric arrangement of the PTVs and the distribution of IDC50% (the transparent yellow cloud around the solid PTVs) as they relate to the individual PTVs. Figure 3(a) shows Plan 1 for five spherical shaped PTVs of widely varying size (volume 0.19–8.0 cm^3^). Figure 3(b) shows Plan 2 for five irregularly shaped PTVs of varying volumes and shapes, some with significant concavity. Figure 3(c) shows Plan 3 for five “jack shaped” PTVs of varying volumes. Clearly, the IDC50% is tightly conformed around the PTV without extraneous intermediate dose distributed between the PTVs. The isolated IDC50% shapes also mirror the shape of their associated PTV, largely like an expanded and slightly smoothed version of the PTV.

The qualitative representation of the AFI approach optimized plans shown in Fig. 3 are summarized quantitatively in Table 4. The PTVs are numbered from patient superior to inferior such that PTV1 is the most superior PTV and PTV5 is the most inferior PTV. The listed M values were generated from Eq. (2), which is the expansion margin of the PTV used to create the OptiForR50shell. Notice that the value of M scales with the size of the PTV. In this case, our R50%_Goal_ came from Eq. (6), and one can easily compare the R50%_Achieved_ for each individual PTV with the R50%_Goal_. Also listed are the final plan quality metrics CI, RTOG Homogeneity Index (HI),^13^ and D_x_% (the % volume of dosimetric coverage of each PTV by the Rx dose). For all 15 PTVs within three plans, a minimum 95% of the volume of each PTV is covered by 100% of the prescription dose, and an average PTV coverage of 96.89% ± 1.65% was obtained. CI and RTOG HI for each PTV within each plan were evaluated individually as shown in Table 4. The average CI and RTOG HI for all 15 PTVs are 1.059 ± 0.060 and 1.314 ± 0.059, respectively.

**Table 4:** The final optimization results of Plans 1–3.

	V_PTV_ (cm^3^)	M (cm)	V_OptiForR50shell_ (cm^3^)	%V_Opti_	R50%_Goal_	R50%_Achieved_	CI	RTOG HI	D_x_%
*Plan 1 (Spherical)*									
PTV1	0.54	0.93	12.05	20	5.43	4.75	1.05	1.409	98.9
PTV2	1.96	1.22	31.96	20	4.20	3.48	1.03	1.341	99.1
PTV3	0.19	0.74	5.50	20	6.69	6.24	1.02	1.221	95.2
PTV4	0.97	1.06	18.99	20	4.83	4.22	1.05	1.373	98.8
PTV5	8.00	1.59	88.10	20	3.17	2.80	0.95	1.338	95.0
	*Total = 11.66*								
*Plan 2 (Irregular)*									
PTV1	1.20	1.10	23.96	18	4.63	4.46	1.09	1.367	98.1
PTV2	0.98	1.06	21.31	18	4.82	4.55	1.00	1.240	95.4
PTV3	0.73	1.00	17.19	17	5.11	4.70	1.03	1.395	95.0
PTV4	1.01	1.07	20.96	18	4.79	4.61	1.05	1.304	97.9
PTV5	1.67	1.18	31.03	18	4.33	4.10	1.01	1.230	96.5
	*Total = 5.59*								
*Plan 3 (Jack)*									
PTV1	1.50	1.16	29.93	17	4.43	4.45	1.11	1.315	98.0
PTV2	1.44	1.15	31.28	16	4.46	4.88	1.19	1.276	98.8
PTV3	1.11	1.09	23.60	17	4.70	4.79	1.09	1.325	95.6
PTV4	1.83	1.20	37.09	16	4.25	4.85	1.09	1.305	96.1
PTV5	1.28	1.12	28.17	16	4.57	5.37	1.14	1.266	95.0
	*Total = 7.16*								

The third column labeled “M” is the PTV expansion margin [Eq. (2)] that created the OptiForR50shell used in the optimization. The fourth column labeled “%V_Opti_” is an optimization parameter given by [Eq. (3)]. Plan 1 (five spherical shaped PTVs) is depicted in Fig. 3(a). Plan 2 (five irregularly shaped PTVs) is depicted in Fig. 3(b). Plan 3 (five jack‐shaped PTVs) is depicted in Fig. 3(c). Notice that the R50%_Achieved_ values are better than the R50%_Goal_ values for all spherical and irregular PTVs.

**Fig. 4:**
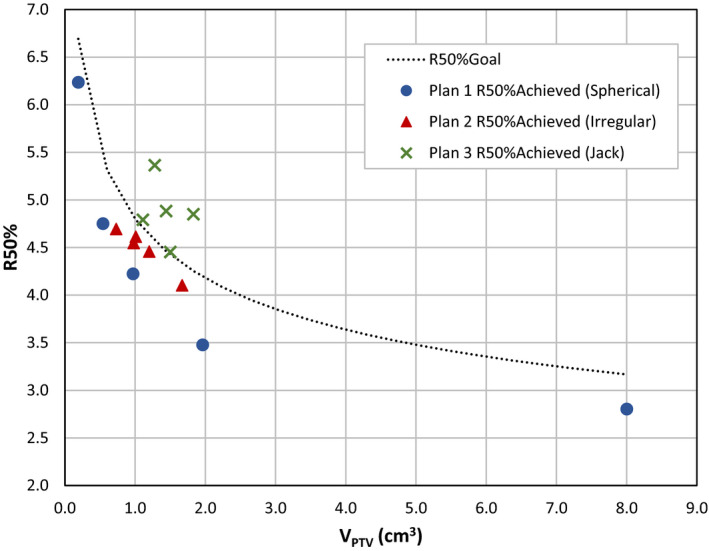
R50%_Achieved_ as a function of PTV volume for each target. The dotted line is the plot of R50%_Goal_ = 4.8/(V_PTV_)^0.2^ [Eq. (6)] extracted from the data of Ballangrud et al. The three data sets represent the individual PTVs of each test plan (Plan 1, spherical PTVs; Plan 2, irregular PTVs; Plan 3, jack PTVs). Improvements in R50% were obtained for all spherical and irregular PTVs as compared to the R50%_Goal_ predicted from the data of Ballangrud et al.

The results for R50% can also be plotted graphically as in Fig. 4. Here, we can easily compare the R50%_Achieved_ for individual PTVs with the R50%_Goal_ as predicted by Eq. (6). Each of the three plans is displayed as a unique data set. In the case of spherical PTVs of any size, the AFI approach optimization yielded R50% values that are clearly better than the R50%_Goal_. The same is true of the irregular shaped PTVs. The jack PTVs exceeded the R50%_Goal_ in most cases, which is not unexpected given the complex nature of these PTVs.

The plan QA passing rates obtained were 97.7% for Plan 1 (spherical PTVs), 98.2% for Plan 2 (irregular PTVs), and 98.2% for Plan 3 (jack PTVs). This indicates that clinically acceptable beam modulation has been generated by the AFI approach and application of the method in a general clinical scenario should not be problematic.

## 4 DISCUSSION

In this work, we address two issues in the simultaneous treatment of multiple cranial lesions: 1. how best to optimize multiple targets independently at the same time, and 2. how to achieve a stated goal for R50% without an iterative approach. We offer an approach that translates the R50%_Goal_ into direct optimizer inputs. Although we do not address the question of the validity of the R50%_Goal_, the values used are obtained from a published treatment planning study. We only claim that, given a reasonable R50%_Goal_, a method to produce optimization parameters that approximately achieve that goal is possible.

Our AFI approach employs a specially constructed optimization shell (OptiForR50shell) around each individual PTV, whose dimensions scale with the size of the individual PTV. One can see in Table 4 that each PTV has a unique expansion margin, M, to construct the OptiForR50shell. Since our benchmark R50%_Goal_ is dependent on the V_PTV_, the expansion margin and thus the OptiForR50shell structures scale with the V_PTV_. The %V_Opti_ is a calculated percent volume input parameter for the optimizer that should be approximately 20% based on the construction of the OptiForR50shell. Since a spherical PTV was assumed in the derivation of M in Eq. (2), the %V_Opti_ values calculated using Eq. (3) will be exactly 20% for spherical PTVs, as can be seen in Table 4, Plan 1. Furthermore, as seen in Table 4, %V_Opti_ values for nonspherical PTVs are less than 20%. PTVs with a higher surface area to volume ratio have %V_Opti_ values smaller than for spherical PTVs. To get an exact value for %V_Opti_, one must explicitly calculate it for every PTV using Eq. (3). Thus, the %V_Opti_ calculation more precisely tunes the optimization parameter to the specific PTV than a simple margin expansion can. This ensures that every PTV in the optimization run has equal priority and that small PTVs do not get lost in the dose statistics of the large PTVs.

In Plan 1, the five spherical PTVs vary widely in size (0.19–8 cm^3^). The rationale for doing this plan was to demonstrate that R50% depends on the size of the PTV and that our approach of simultaneously optimizing PTVs of widely varying sizes is successful as demonstrated in Fig. 3(a).

The R50% values achieved in an optimization for the multiple target test plans, R50%_Achieved_, are listed along with the R50%_Goal_ for each PTV individually in Table 4. Those R50% values are plotted in Fig. 4 where one can see R50%_Achieved_ is less than R50%_Goal_ for all the spherical and irregularly shaped PTVs. The jack‐shaped PTVs have R50%_Achieved_ values that are generally larger than the R50%_Goal_, which is based only on PTV volume. We contend that larger R50%_Achieved_ values for jacks result from the anomalously high PTV surface area of these jack PTVs, much as demonstrated in Desai et al. for lung SBRT targets.^21^

It is clear from published clinical data that different PTV characteristics, such as PTV volume, have an impact on the R50% value one could expect to achieve, R50%_Goal_. Because this R50%_Goal_ is clearly dependent on PTV characteristics, a one‐size‐fits‐all approach to optimization dose limiting shells will be less effective at achieving the defined benchmark R50%_Goal_. Our AFI approach is an empirically derived, custom‐tailored approach that adapts to the individual PTV and yields the needed direct inputs for the inverse planning optimizer to achieve the stated R50%_Goal_. The AFI approach translates the R50%_Goal_ into the needed optimizer inputs. If improved estimates for R50%_Goal_ emerge, the AFI approach will change the OptiForR50shell characteristics and provide the needed optimizer inputs for the updated R50%_Goal_.

The scaling of the OptiForR50shell dose control shell to the specific characteristics of each individual PTV is an important new aspect of this work. Other authors have used dose control shells, but those shells are always uniform expansions of the PTVs regardless of the individual PTV characteristics (volume, surface area, etc.). As such, one cannot apply the same constraints uniformly to all PTV variants and get the optimal solution for each individual PTV. Using the AFI approach, we were able to ensure that each PTV is given equal priority in the optimizer. Assigning equal proprieties to each PTV and equal priorities to the OptiForR50shell ensures good dose drop off without compromising PTV coverage. Dose drop‐off is critical in a multiple metastasis case since the justification is to avoid whole brain irradiation.

Several prior works have discussed how the final planning goal as determined from a single target SRS/SRT can be applied to a multiple target case.^22^, ^23^ Indeed, Goldbaum et al. specifically state that a recent study generated a predictive model of V12 for single‐target single‐isocenter SRS delivery using only DCAT plan data and showed that it could be accurately applied to dosimetric prediction of multi‐target single‐isocenter VMAT plans. There are numerous proposed standards for what the final R50%_Goal_ should be, and the results for studies that focus on a single target could be relevant to a multiple target case. Again, regardless of the stated R50%_Goal_ the AFI approach is adaptable and will yield R50% values that are close to those goals or, as shown in this work, sometimes even R50% values that are less than the R50%_Goal_ can be obtained.

Because each OptiForR50shell surrounds its respective PTV and touches the PTV outer surface, the OptiForR50shell also provides a convenient analysis tool for the optimization of the PTV within the shell. One can assess the high dose spill from an individual PTV by taking the ratio of the volume of the OptiForR50shell that has 105% of the Rx dose to V_PTV_. This is the V105% metric common in Lung SBRT. One can directly measure the volume of the IDC50%shell by simply finding the volume of OptiForR50shell that is within the 50% isodose cloud, and thus, the final R50% for each individual PTV can be computed from Eq. (11). Noting that R50% = CI × GI, this one metric encapsulates both CI and GI common in SRS/SRT into one metric. To extract the CI directly for each PTV, one needs to use Eq. (9), which involves only the commonly reported dose data for a given PTV and its corresponding OptiForR50shell.

This study was conducted in a phantom with well‐spaced targets, and no consideration was given to other critical structures such as the brainstem. A potential advantage of the AFI approach is the ability to accommodate the asymmetric intermediate dose flair that happens when a particular PTV is in close proximity to a critical structure that must be spared from the intermediate dose – such as the optic chiasm or brainstem. Because the AFI approach is designed to generate OptiForR50shell structures that are only 20% occupied by the IDC50%shell, the OptiForR50shell is relatively large, and thus, the dose pushed out of the nearby critical structure can still be contained within the OptiForR50shell but asymmetrically within the OptiForR50shell. Based on application of the conservation of integral dose hypothesized by Reese et al.,^24^ the R50%_Goal_ need not change because of the adjacent critical structure, and the OptiForR50shell can still contain the IDC50%shell. Another potential problematic situation may occur when two PTVs are near each other. In this situation, the OptiForR50shell structures may overlap, and the resulting performance of the AFI approach is uncertain. One possible modification for nearly coincident PTVs is to combine these into a single PTV and proceed as described previously. Further study is required to evaluate the performance of the AFI approach in these more demanding clinical situations.

The AFI method was developed within a particular set of conditions, those being LINAC based VMAT delivery and the Varian Eclipse TPS. However, the approach is expected to be portable to other treatment delivery platforms and treatment planning and optimization systems. The derivation of the expansion margin, M, used to construct OptiForR50shell, and the optimizer input parameter %V_Opti_ are based on physical principles that are likely universal. However, it is possible that some minor modifications, such as objective penalties, may be necessary in other technologies.

As noted in Section 2.A, the AFI strategy can accommodate any stated R50%_Goal_. Desai et al. derive a theoretical value for R50%_Goal_, which they name R50%_Analytic_ ^25^ R50%_Analytic_ is explicitly dependent on the volume and surface area of the PTV. Thus, using R50%_Goal_ = R50%_Analytic_ in the AFI strategy would allow one to incorporate the PTV surface area into the optimization criterion.

While this is not the only successful approach for achieving R50% goals, this is one quantitative method that allows the planner to prospectively customize the optimization structures and parameters based on the R50% goal for a multi‐target, single isocenter plan. The potential for reducing the treatment planning time in these cases may be important in a busy radiation therapy clinic. Additionally, the AFI strategy could be built into a knowledge‐based optimization system, which could be particularly powerful if one used R50%_Analytic_ as R50%_Goal_.^25^ A future comparison of the AFI strategy with current NTO‐driven optimizations (such as HyperArc) or knowledge‐based techniques (such as RapidPlan) could also prove fruitful.

## 5 CONCLUSION

One can prospectively determine the size of an optimization structure, OptiForR50shell, around a given PTV for an assumed planning goal for R50% when treating multiple cranial PTVs using a single isocenter. Methodology has been presented that defines the optimization structure geometry and optimization objectives based on V_PTV_ and the R50%_Goal_. Successful implementation of the AFI approach has been demonstrated for treatment planning and optimization on the Eclipse TPS and treatment delivery via Varian TrueBeam RapdiArc VMAT technology. The technique is considered to be portable to other treatment planning systems and treatment delivery platforms with little or no modification.

## CONFLICT OF INTEREST

No conflicts of interest.

## Funding

There are no funders to report for this submission.

## Data Availability

The data that support the findings of this study are available from the corresponding author upon reasonable request.

## APPENDIX A ABBREVIATIONS

Table A1 contains definitions for abbreviations used throughout this article.

**Table A1:** List of abbreviations with definitions.

Abbreviation	Definition
AFI	“Ask For It” methodology; uses R50%_Goal_ value to determine optimization parameters
CI	Conformity index; V_IDC100%_ divided by V_PTV_
DCAT	Dynamic conformal arc therapy
DVH	Dose volume histogram
Dx%	Percent volume of dosimetric coverage of each PTV by the prescription dose
GI	Gradient index; V_IDC50%_ divided by V_IDC100%_
HI	Homogeneity index
IDC	Isodose cloud
IDC50%	50% (of prescription dose) isodose cloud
IDC50%shell	Distance from the edge of the planning target volume to the edge of the 50% isodose cloud
IDC100%	100% (of prescription dose) isodose cloud
iShell	Insurance shell; insures the 50% isodose cloud remains within each individual OptiForR50shell
M	Expansion margin; margin around the PTV needed to create OptiForR50shell
NTO	Normal tissue objective; instructs the optimizer to limit dose to nontarget volumes
OAR	Organs at risk
OptiForR50	Structure created by expanding the PTV by the margin M
OptiForR50shell	Structure created by subtracting the PTV from OptiForR50
OptiForR50shelln	OptiForR50shell for the n^th^ PTV
PTV	Planning target volume
PTV_n_	The n^th^ planning target volume
r_PTV_	Radius of the planning target volume
R50%	Ratio of the volume of the 50% isodose cloud to the volume of the planning target volume
R50%_Achieved_	Value of R50% obtained after treatment planning is complete
R50%_Goal_	Target value of R50% used in plan optimization
SBRT	Stereotactic body radiotherapy
SRS	Stereotactic radiosurgery
SRT	Stereotactic radiotherapy
TPS	Treatment planning system
V_IDC50%_	Volume of the 50% isodose cloud
V_IDC50%shell_	Volume of the 50% isodose cloud minus the volume of the planning target volume
V_IDC100%_	Volume of the 100% isodose cloud
V_OptiForR50shell_	Volume of the OptiForR50shell structure
V_OptiForR50shelln_	Volume of the OptiForR50shell structure for PTV_n_
V_PTV_	Volume of the planning target volume
V_PTVn100%_	Volume of the 100% prescription dose that covers PTV_n_
VMAT	Volumetric modulated arc therapy
%V100%_OptiForR50shelln_	Percent of the volume of OptiForR50shell for PTV_n_ that receives 100% of the prescription dose
%V100%_PTVn_	Percent of the volume of PTV_n_ that receives 100% of the prescription dose
%V50%_OptiForR50shell_	Percent of the volume of OptiForR50shell that receives 50% of the prescription dose
%V_Opti_	Percent of the volume of OptiForR50shell that occupies 50% or less of the prescription dose

## APPENDIX B DERIVATION OF EXPRESSION FOR M

### B.1 Definition of V_OptiForR50shell_

Consider the simplified anatomy of an optimization structure OptiForR50shell and its relationship to IDC50%shell and PTV as illustrated in Fig. 1. To minimize the value for R50%, one must reduce the size of IDC50%shell. The size of the IDC50%shell can be minimized within the framework of the Eclipse TPS by reducing its size within the structure OptiForR50shell. For this, we need to know the volume occupied by IDC50%shell within the volume of OptiForR50shell. To begin, we define %V_Opti_ as the percentage of the OptiForR50shell occupied by IDC50%shell as represented in fractional terms by the following equation:

(B1) $$\%V_{\text{Opti}\;}=\frac{V_{\text{IDC50}\%\text{shell}}}{V_{\text{OptiForR50shell}}}\times 100$$

From the basic definition of VIDC50%shell, we know:

(B2) $$V_{\text{IDC50}\%\text{shell}\;}=V_{\text{IDC50}\%}‐V_{\text{PTV}}$$

The substitution of Eq. (B2) for V_IDC50%shell_ into Eq. (B1) gives:

(B3) $$\%V_{\text{Opti}}=\frac{{\text{(V}}_{\text{IDC50}\%}‐V_{\text{PTV}})}{V_{\text{OptiForR50shell}}}\times 100$$

By factoring V_PTV_ out of the numerator of Eq. (B3), the resulting equation is:

(B4) $$\%V_{\text{Opti}\;}=\frac{\left(\frac{V_{\text{IDC50}\%}}{V_{\text{PTV}}}‐1\right){\;V}_{\text{PTV}}}{V_{\text{OptiForR50shell}}}\times 100$$

The following equation is the definition of R50%:

(B5) $$\text{R50\textbackslash{}\% =}\;\frac{V_{\text{IDC50\textbackslash{}\%}\;}}{V_{\text{PTV}}}={\text{R50\textbackslash{}\%}\;}_{\text{Goal}}$$

The substitution of Eq. (B5) into Eq. (B4) gives:

(B6) $$\%V_{\text{Opti}\;}=\frac{\text{(R50}{\%}_{\text{Goal}}‐{\text{1) V}}_{\text{PTV}}}{V_{\text{OptiForR50shell}}}\times 100$$

Equation (B6) expresses the percentage of the volume V_OptiForR50shell_ occupied by V_IDC50%shell_ in terms of the value of R50%Goal.

Quantitatively, the only unknown value in Eq. (B6) is the volume of OptiForR50shell (V_OptiForR50shell_). We know the optimization structure OptiForR50shell must be larger than IDC50%shell but how much larger is uncertain. Therefore, V_OptiForR50shell_ is expressed in the following symbolic terms where N is a real number larger than 1:

(B7) $$V_{\text{OptiForR50shell}}={\text{N (V}}_{\text{IDC50}\%\text{shell}})$$

### B.2 Form 1 of V_OptiforR50shell_

By combining Eqs. (B5) and (B2), the following equation for R50% in terms of V_IDC50%shell_ is given:

(B8) $$\text{R50}{\%}_{\text{Goal}}=\frac{{\text{(V}}_{\text{IDC50}\%})}{V_{\text{PTV}}}=\frac{{\text{(V}}_{\text{IDC50}\%\text{shell}}+V_{\text{PTV}})}{V_{\text{PTV}}}=\frac{V_{\text{IDC50}\%\text{shell}}}{V_{\text{PTV}}}+1$$

Further algebraic manipulation of Eq. (B8), yields:

(B9) $$V_{\text{IDC50}\%\text{shell}}=\text{(R50}{\%}_{\text{Goal}}‐{\text{1) V}}_{\text{PTV}}$$

The substitution of Eq. (B9) into Eq. (B7) gives an expression for VOptiForR50shell in terms of R50%.

(B10) $$V_{\text{OptiForR50shell}}=N\;\left(\text{R50}{\%}_{\text{Goal}}‐1\right)V_{\text{PTV}}$$

### B.3 Form 2 of V_OptiForR50shell_

The optimization structure OptiForR50shell is generated by uniformly expanding PTV by some margin M (Fig. 1) and subtracting PTV from the resulting structure. The volume of the generated OptiForR50shell structure is then expressed as:

(B11) $$V_{\text{OptiForR50shell}}=V_{\text{(PTV + M)}}‐V_{\text{PTV}}$$

where (PTV + M) represents an operation of expanding PTV by some margin M.

For the sake of simplicity, the PTV is assumed to be of a spherical shape, and the volume of the PTV is expressed as:

(B12) $$V_{\text{PTV}}=\frac{4}{3}{\pi r}_{\text{PTV}}^{3}$$

where r_PTV_ is the radius of the PTV.

Thus, V_OptiForR50shell_ in Eq. (B11) is expressed in algebraic terms as:

(B13) $$V_{\text{OptiForR}50\text{shell}}=\frac{4}{3}\pi {\left(r_{\text{PTV}}+M\right)}^{3}‐\frac{4}{3}\pi r_{\text{PTV}}^{3}$$

### B.4 Expression for M

Equations (B13) and (B10) are two different forms of V_OptiForR50shell_ that lead to an expression for M as a function of R50%_Goal_. To begin, the two equations are set equal:

(B14) $$\frac{4}{3}\pi {\left(r_{\text{PTV}}+M\right)}^{3}‐\frac{4}{3}\pi r_{\text{PTV}}^{3}=N\left(R50{\%}_{\text{Goal}}‐1\right)V_{\text{PTV}}$$

By solving Eq. (B14) for M, Eq. (B15) results:

(B15) $$M=r_{\text{PTV}}\text{[\textbackslash{}\{ N}\left(\text{R50}{\%}_{\text{Goal}}‐1\right)+{\text{1\textbackslash{}\}}\;}^{1/3}‐1]$$

Alternatively, r_PTV_ can be expressed in terms of V_PTV_, which yields:

(B16) $$M={\left(\frac{3}{4\pi }V_{\text{PTV}}\right)}^{1/3}\left[{\left\{N\left(R50{\%}_{\text{Goal}}‐1\right)+1\right\}}^{1/3}‐1\right]$$

Equation (B16) is a general equation for M and indicates how M is proportional to both V_PTV_ and R50%. For the specific case where V_OptiForR50shell_ is chosen to be five times larger than V_IDC50%shell_, Eq. (B16) reduces to:

(B17) $$M={\left(\frac{3}{4\pi }V_{\text{PTV}}\right)}^{1/3}\left[{\left(5\times R50{\%}_{\text{Goal}}‐4\right)}^{1/3}‐1\right]$$

Equation (B17) is the form of M used in the “Ask For It” (AFI) approach described in the Methods section.
